# Population Pharmacokinetics of Cyclosporine in Chinese Pediatric Patients With Acquired Aplastic Anemia

**DOI:** 10.3389/fphar.2022.933739

**Published:** 2022-07-26

**Authors:** Xuan Gao, Zhu-Li Bian, Xiao-Hong Qiao, Xiao-Wen Qian, Jun Li, Guo-Mei Shen, Hui Miao, Yi Yu, Jian-Hua Meng, Xiao-Hua Zhu, Jun-Ye Jiang, Jun Le, Ling Yu, Hong-Sheng Wang, Xiao-Wen Zhai

**Affiliations:** ^1^ Outpatient and Emergency Management Office, National Children’s Medical Center, Children’s Hospital of Fudan University, Shanghai, China; ^2^ Department of Pediatrics, Tongji Hospital, School of Medicine, Tongji University, Shanghai, China; ^3^ Department of Hematology and Oncology, National Children’s Medical Center, Children’s Hospital of Fudan University, Shanghai, China

**Keywords:** cyclosporine, population pharmacokinetics, NONMEM, acquired aplastic anemia, pediatric patients

## Abstract

Cyclosporine (CsA) is a component of the first-line treatment for acquired aplastic anemia (acquired AA) in pediatric patients. This study aimed to develop a population pharmacokinetic (PK) model of CsA in Chinese pediatric patients with acquired AA to inform individual dosage regimens. A total of 681 CsA whole blood concentrations and laboratory data of 157 pediatric patients with acquired AA were retrospectively collected from two hospitals in Shanghai. A nonlinear mixed-effect model approach was used to build the population PK model. Potential covariate effects of age, body weight, and biochemical measurements (renal and liver functions) on CsA PK disposition were evaluated. Model fit was assessed using the basic goodness of fit and a visual predictive check. The CsA concentration data were accurately described using a two-compartment disposition model with first-order absorption and elimination. Body weight value was implemented as a fixed allometric function on all clearance and volume of distribution parameters. Total bilirubin level was identified as a significant covariate on apparent clearance (CL/F), with a 1.07% reduction per 1 nmol/L rise in total bilirubin level. The final estimates for CL/F and central volume (Vc/F) were 29.1 L/h and 325 L, respectively, for a typical 28 kg child. Other covariates (e.g., gender, age, albumin, hemoglobin, hematocrit, serum creatinine, and concomitant medication) did not significantly affect the PK properties of CsA. This population PK model, along with a maximum a *posteriori* Bayesian approach, could estimate individual PK parameters in pediatric patients with acquired AA to conduct individual CsA therapy.

## Introduction

Acquired aplastic anemia (acquired AA) is a rare heterogeneous disorder characterized by peripheral pancytopenia and bone marrow aplasia or hypoplasia. Most patients with acquired AA (70–80%) are idiopathic because their primary etiology remains unknown (Marsh et al., 2009; Shallis et al., 2018). The annual incidence of acquired AA in Asians is 2- to 3-fold higher than that in the Western population, which has been reported to be approximately 2–2.3 patients per million (Issaragrisil et al., 1991; Montane et al., 2008; Young and Kaufman, 2008). The median age at disease diagnosis among children is around 9 years (Jeong et al., 2011). Acquired AA diagnosis was divided into three subtypes according to the related clinical guidance (Camitta et al., 1975; Camitta et al., 1976; Bacigalupo et al., 1988; Brodsky and Jones, 2005), including nonsevere AA (NSAA), severe AA (SAA), and very severe AA (vSAA). As per current clinical guidelines, immunosuppressive therapy (IST) using antithymocyte globulin (ATG) combined with cyclosporine A (CsA) is the standard first-line treatment for patients with SAA or vSAA without a suitable donor and for those with NSAA who are transfusion-dependent or experienced bleeding (Speck et al., 1977; Bacigalupo et al., 1988; Marsh et al., 1999; Rosenfeld et al., 2003; Viollier et al., 2005; Yoshida and Kojima, 2018). The overall survival rate of patients treated with IST was reported to be 68–90%, and the response rate ranged from 58 to 90% (Frickhofen et al., 1991; Rosenfeld et al., 1995; Frickhofen and Rosenfeld, 2000; Rosenfeld et al., 2003; Führer et al., 2005; Locasciulli et al., 2007; Pongtanakul et al., 2008; Saracco et al., 2008; Deyell et al., 2011; Samarasinghe et al., 2012; Dufour et al., 2015).

CsA, a classic calcineurin inhibitor, has been widely used in IST for decades (Yoshida and Kojima, 2018). CsA is mainly metabolized *via* cytochrome P450 isoenzymes (CYP) 3A4 and 3A5 in the liver and is also a substrate of P-glycoprotein (Kronbach et al., 1988; Aoyama et al., 1989; von Richter et al., 2004; Wojnowski, 2004; Patel and Wairkar, 2019). CsA has been reported to have high variability in its pharmacokinetic (PK) disposition (Ptachcinski et al., 1986), especially for oral dosing (Lindholm et al., 1988; Patel and Wairkar, 2019). CsA has a narrow therapeutic window for immunosuppressive purposes, usually with a whole blood trough concentration of 100–200 ng/ml for clinical indications (Marsh et al., 2009; Jain et al., 2019). Suboptimal concentration results in an insufficient clinical response, and high exposure raises patient safety concerns. The serious adverse effects included dyslipidemia, posttransplant diabetes mellitus, hypertension, intermittent renal hypoperfusion, and both reversible acute toxicity and irreversible tubulointerstitial fibrosis (Dunn et al., 2001; Olyaei et al., 2001). Its effects are different in children compared to adults because of the developmental processes, and the ontogeny of enzymes and body size could affect the disposition of the drug in the body. Thus, according to clinical guidelines, routine therapeutic drug monitoring (TDM) is strongly recommended for CsA, particularly in pediatric patients.

Population PK properties of CsA in pediatric patients have been investigated in several clinical trials, mainly targeting stem cell transplantation, posttransplantation, and nephrotic syndrome conditions (Irtan et al., 2007; Willemze et al., 2008; Kim et al., 2015; Li et al., 2019; Zhao et al., 2022). To date, population PK analysis of CsA in children with acquired AA has seldom been reported (Ni et al., 2013). Considering the remarkable difference in the physiopathology between transplantation and acquired AA, PK extrapolation across indications would have high uncertainties. This study aimed to establish a population model to characterize the PK of CsA in Chinese pediatric patients with acquired AA and to explore the potential covariate effects. The proposed population PK model can provide guidance for individual CsA therapy in pediatric patients with acquired AA.

## Materials and Methods

### Study Population

The eligibility criteria for the study population were pediatric patients (≤18 years old) diagnosed with acquired AA who received CsA treatment at two hospitals in Shanghai (Children’s Hospital of Fudan University and Tongji Hospital of Tongji University) from January 2014 to December 2021. The diagnosis of acquired AA was based on several critical criteria (e.g., peripheral blood investigations, bone marrow smear, and biopsy) and excluded other disease conditions such as autoimmune disease, congenital bone marrow failure, acute myeloid leukemia, acute lymphoblastic leukemia, myelodysplastic syndrome, and other malignant hematological tumors. Patients with acquired AA who received stem cell transplantation were also excluded from this study. The study protocol and the data collection were approved by the hospital’s research ethics committee.

### Acquired Aplastic Anemia Treatment Protocol

The acquired AA treatment regimen was in accordance with the suggestions in the clinical guidance, including IST-containing treatment (e.g., ATG + CsA, CsA + androgen, or CsA monotherapy) and supportive care measures (transfusions, protective isolation, antibiotics, and others). The initial oral dosing regimen of CsA was 5 mg/kg/day orally, twice daily. In general, the dose was adjusted from 5 to 8 mg/kg/day and could even to 10 mg/kg/day, depending on CsA concentration, to ensure that the concentration is within the therapeutic window. As per a clinical guideline for childhood acquired AA in China (The Society of Pediatrics, 2014), it was recommended that the therapeutic windows for CsA be 100–200 ng/ml and 300–400 ng/ml for trough and peak concentration, respectively. The first whole blood concentration of CsA at a steady state was monitored after 2 weeks of administration, and then every 3–6 months afterward, if indicated. The dose was reduced only after the concentration was maintained at these levels for at least 12 months. The dose was tapered slowly (e.g., 10–20% of the original dosage was tapered once every 3 months). The clinical physician closely monitored the complete blood count, liver and renal functions, and whole blood CsA concentration at each time of dose adjustment and carefully reduced the amount if there was any fluctuation. All treatments were in accordance with the diagnosis and treatment recommendations of the pediatric society of the Chinese Medical Association.

### Pharmacokinetic Sampling and Measurement

For TDM purposes, only sparse whole blood samples were routinely collected for CsA concentration measurement according to clinical practice in the two study hospitals. The PK samples were collected at or around the peak (2–4 h postdosing) and predose (within 1 h prior to dosing). CsA whole blood concentrations were determined using an Emit® 2000 Cyclosporine Specific assay (6R079UL; Siemens Healthcare Diagnostics, Inc., Newark, NJ, United States) in accordance with the procedures in the manual.

### Data Collection

All relevant clinical data for the present population PK analysis were collected from patients’ medical records in the hospital information system. The data mainly contained: 1) Demographic data (gender, age at treatment, and body weight at treatment); 2) laboratory tests, including but not limited to complete blood count (red blood cell, white blood cell, platelets, hemoglobin [Hb], hematocrit [HCT]), liver function (aspartate transaminase [AST], alanine transaminase [ALT], alkaline phosphatase [ALP], direct bilirubin, total bilirubin [TBIL]), and renal function (serum creatinine [SCr] levels); 3) concomitant medications (e.g., granulocyte colony-stimulating factor, rabbit ATG [r-ATG], glucocorticoids, and testosterone undecanoate) during the therapy; and 4) PK data, such as the date and time of CsA administration, and CsA concentration readout.

### Population Pharmacokinetic Analysis Approach

Population PK analysis was conducted using a nonlinear mixed-effect modeling approach using NONMEM® software (version 7.4, ICON Development Solutions, Ellicott City, MD, United States) with a gFortran compiler (version 4.6.0). PsN (version 4.6.0) and the R language (version 3.4.0) were used to summarize and visualize the modeling outputs. First-order conditional estimation with the η-ε interaction algorithm (FOCE-I) was utilized throughout the model-building procedures. Discrimination between hierarchical models was based on the objective function value (OFV), which was proportional to twice the log-likelihood (-2LL). A decrease in OFV (∆OFV) of 3.84 was considered a statistically significant improvement in model fitting (*p* < 0.05) between the two hierarchical models after the inclusion of one additional parameter (*df* = 1).

CsA concentrations were logarithmically converted for modeling analysis. A base model was selected without any covariates capable of appropriately capturing the concentration–time data. During the base model selection stage, all possible structural compartments (i.e., one- and two-compartment disposition models) were investigated.

Interindividual variability (IIV) was modeled as an exponential function on all PK parameters, where applicable (Eq. 1).

$$\theta_{i}=\;\theta •\text{exp}\left(\eta_{i,\theta }\right)$$ (1)

where $\theta_{i}$ is the individual PK parameter estimate for the *i*th individual patient, θ is the population estimate of the investigated PK parameter, and $\eta_{i,\theta }$ is the IIV of the investigated PK parameter, which is assumed to follow a normal distribution with a zero mean and variance ω^2^. The residual variability, assumed to be normally distributed with zero mean and variance σ^2^, was modeled with an additive error on the natural log-transformed concentrations, which was approximately equal to an exponential residual error on an arithmetic scale.

### Covariate Modeling

Body weight was implemented in the model as a simultaneous inclusion of an allometric function for all clearance and distribution volume parameters (Eqs 2, 3, respectively).

$$CL_{i}=\;CL_{typical}\cdot {\left(\frac{BW_{i}}{BW_{median}}\right)}^{0.75}\cdot \exp\left(\eta_{i,CL}\right),$$ (2)

$$V_{i}=\;V_{typical}\cdot \left(\frac{BW_{i}}{BW_{median}}\right)\cdot \exp\left(\eta_{i,V}\right),$$ (3)

where BW_i_ is the individual body weight for the *i* ^th^ individual and BW_median_ is the median body weight of the study population (28 kg).

In addition to body weight, other potential covariates, such as age, gender, laboratory tests of liver and renal function, and concomitant drugs, were investigated for all model parameters, except absorption-rate constant, using a forward selection (*p* = 0.05) and followed a strict backward elimination (*p* = 0.01) procedure.

### Model Evaluation

Basic goodness-of-fit plots, such as the conditional weighted residue versus population prediction, conditional weighted residue versus time, observation versus population prediction, and individual prediction, were used to evaluate systematic discrepancies and model misspecification if it exists. The sampling importance resampling approach was employed to derive parameter uncertainties for the final population PK model with the options of sample = 2,000 and resample = 1,000. The overall predictive performance of the final population PK model was evaluated using prediction-corrected visual predictive checks [(Bergstrand et al., 2011), n = 1,000 simulations].

### *In Silico* Simulation

Based on the final population PK model, *in silico* simulations were conducted according to different clinical scenarios, such as body weight, significant covariates, and dosing regimens (*n* = 1,000 for each scenario). The simulated PK exposure parameters (e.g., trough concentration) for each scenario were summarized and visualized.

## Results

In total, 681 whole blood CsA concentrations of samples from 157 pediatric patients were included in the current population PK modeling analysis. The basic demographic characteristics of patients are presented in Table 1. Overall, the baseline demographic data of the patients were comparable between the two hospitals.

**TABLE 1:** Demographic data of children with acquired aplastic anemia.

Variable	Value
Patient numbers	157
Age (years) [median (range)]	7.8 (1.5, 18.8)
Bodyweight (kg) [median (range)]	27.5 (12.0, 91.0)
The severity of AA, *n* (%)	
NSAA	94 (59.9)
SAA	40 (25.4)
vSAA	21 (13.4)
Biochemistry tests [median (range)]	
White blood cell (10^9^/L)	3.97 (0.26, 12.10)
Hb (g/ml)	85 (48.2, 150)
Neutrophil (%)	34.4 (1.1, 83.0)
Blood urea nitrogen (mmol/L)	5.10 (1.10, 16.30)
Total protein (g/L)	66.8 (45.5, 86.6)
Albumin (g/L)	40.3 (25.8, 48.4)
ALT	13.0 (1.0, 209.9)
AST	21.0 (8.0, 91.3)
Total bilirubin (µmol/L)	10.90 (4.00, 70.00)
Direct bilirubin (µmol/L)	2.50 (0.30, 39.50)
Serum creatine (µmol/L)	40.0 (16.0, 127.0)
Concomitant medications, *n* (%)	
Testosterone undecanoate	111 (70.7)
Prednisone	24 (15.3)
Methylprednisolone	47 (29.9)
Prednisolone	2 (1.3)
Dexamethasone	1 (0.6)
r-ATG	47 (29.9)
Granulocyte colony-stimulating factor	27 (17.2)

Note: The continuous variables were presented as median (range). NSAA, nonsevere aplastic anemia; SAA, severe aplastic anemia; vSAA, very severe aplastic anemia; Hb, hemoglobin; ALT, alanine transaminase; AST, aspartate transaminase; r-ATG, rabbit anti-thymocyte globulin.

A one-compartment model with first-order absorption and elimination processes offered an OFV of 299.011. Utilizing a two-compartment model indicated a significant improvement in the model fit (∆OFV = −249.597). However, the peripheral volume of distribution estimate was implausible (6350 L); therefore, this parameter and intercompartment clearance were fixed at 496 and 5 L/h, respectively, according to the reported literature value (Eljebari et al., 2012). This model still resulted in a superior model fit compared with the one-compartment model (∆OFV = −136.676).

Body weight implemented as an allometric function on all clearance and volume of distribution parameters in the model did not lead to a worse model (∆OFV = −1.363).

Further inclusion of albumin (ALB) on clearance with a linear function resulted in a significant decrease in OFV (∆OFV = −11.425). However, the parameter estimate had poor precision (RSE = 56%); therefore, ALB was not retained in the model. Inclusion of TBIL on clearance in a linear manner led to a significant improvement in the model fit (∆OFV = −8.762) with a good precision of estimate (RSE = 20.4%) and therefore was retained in the model. Other covariates had no statistically significant effects on PK parameters.

**FIGURE 1:**
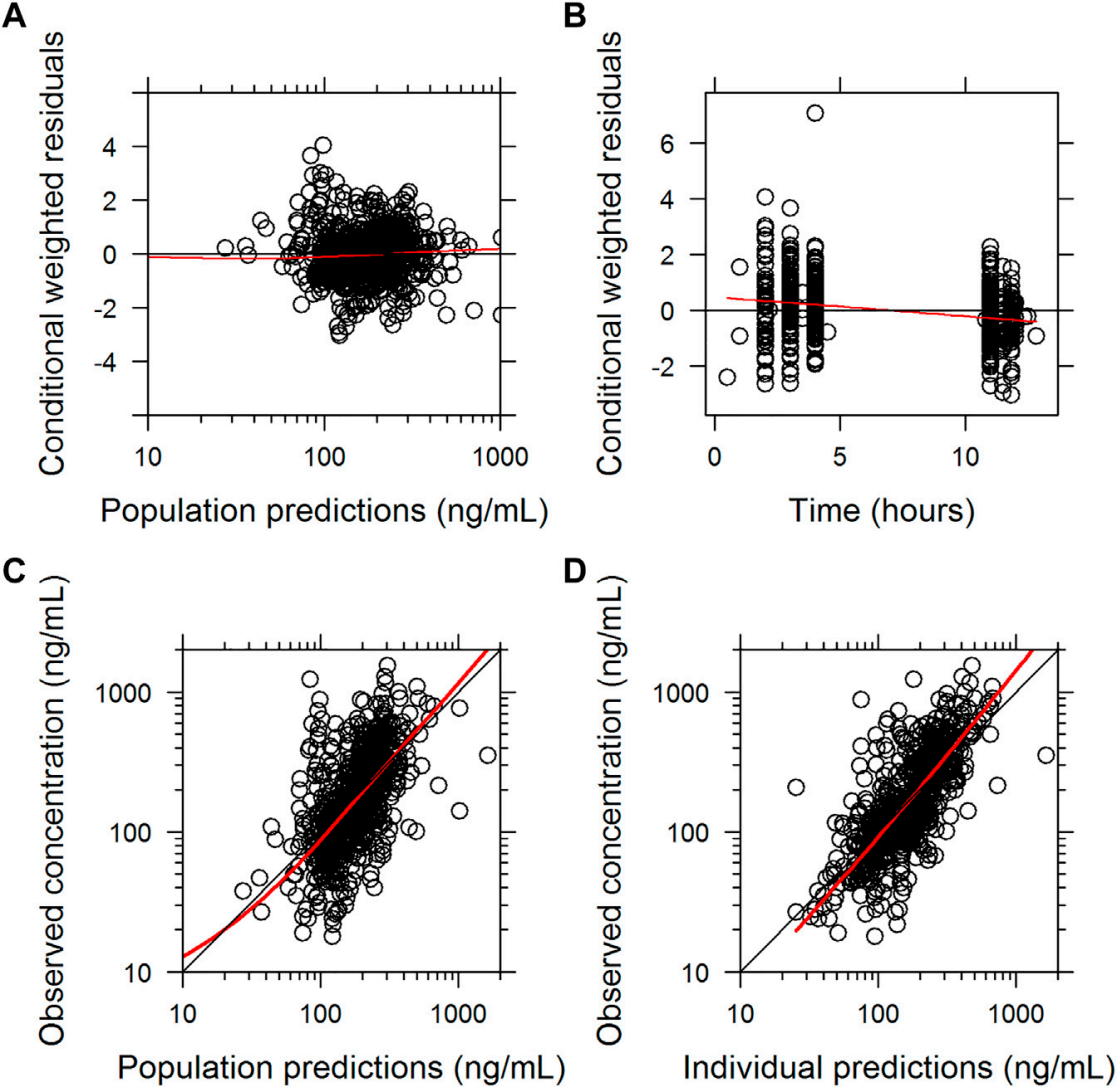
Basic goodness of fit of the final population pharmacokinetic model of cyclosporine A. **(A)** conditionally weighted residuals vs. population-predicted concentrations**. (B)** conditionally weighted residuals vs. time. **(C)** observed plasma concentrations vs. population-predicted concentrations. **(D)** observed plasma concentrations vs. individually predicted concentrations; solid red lines represent locally weighted least-squares regressions.

**FIGURE 2:**
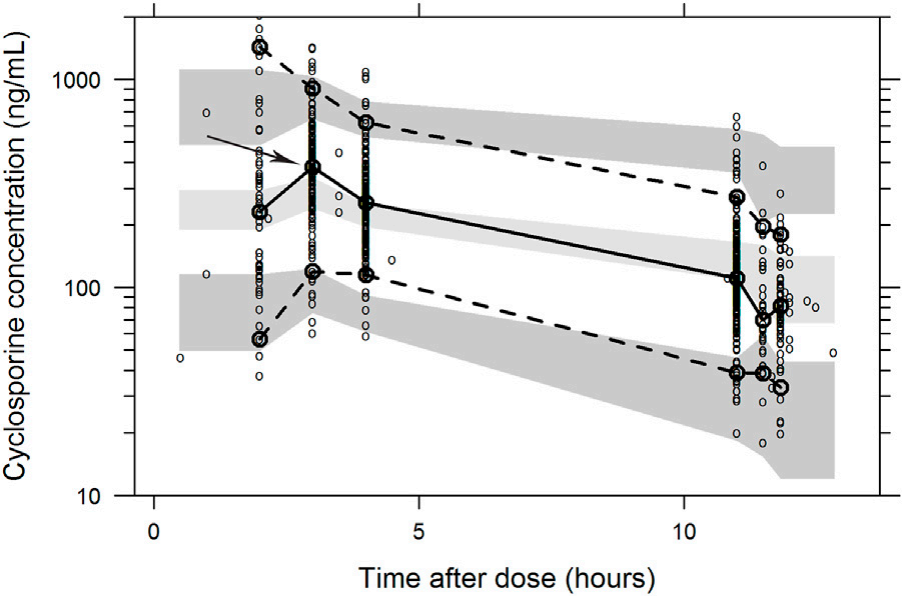
Visual predictive check of the final population pharmacokinetic model of cyclosporine A. The visual predictive check was based on 1,000 stochastic simulations. Open circles are the observations and solid lines represent the 5^th^, 50^th^, and 95^th^ percentiles of the observed data. The shaded areas represent 95% prediction intervals around the simulated 5^th^, 50^th^, and 95^th^ percentiles. The Cmax was slightly underestimated.

The final parameter estimates had a good precision (RSE<30%) and confirmed the stability of the model (Table 2). The basic goodness-of-fit diagnostic plots (Figure 1) did not show any evident systematic discrepancies. Overall, the predictive-corrected visual predictive checks showed good consistency between the model-predicted and observed CsA concentration versus time profiles, although the maximum concentrations were slightly underestimated (Figure 2).

**TABLE 2:** Pharmacokinetic parameter estimates from the final population model of cyclosporine A in children with acquired aplastic anemia.

Parameters	NM estimates	SIR median (95%CI)	CV for IIV	SIR median (95%CI)	Shrinkage (%)
Ka (/h)	1.26 (20.9)	1.27 (0.78–2.02)	—	—	—
CL/F (L/h)	29.1 (3.8)	29.0 (26.9–31.2)	28.0 (24.9)	28.7 (20.9–35.7)	34.7
V_C_/F (L)	325 (15.7)	319 (273–398)	62.1 (29.3)	62.1 (48.7–77.3)	43.6
Q/F (L/h)	3.1 FIX	—	—	—	—
V_P_/F—(L)	262 FIX	—	—	—	—
TBIL on CL (%)	−1.07 (20.4)	−1.05 (−1.50 to −0.50)	—	—	—
σ	0.348 (10.1)	0.348 (0.356–0.393)	—	—	—

Ka is the first-order absorption-rate constant. CL/F represents the apparent elimination clearance. V_C_/F is the apparent central volume of the distribution. Q/F is the apparent intercompartmental clearance. V_P_/F is the apparent peripheral volume of the distribution. σ is the additive residue error on the logarithmic scale. Population estimates in Table 2 are given for a “typical” child with a body weight of 28 kg. Body weight was implemented as a fixed allometric function on all clearance and volume of distribution parameters using a power coefficient of 0.75 and 1.0, respectively. The coefficient of variation for interindividual variability (IIV) was calculated as 100 × (e^variance^)^1/2^. The relative standard error (%RSE) was calculated as 100 × (standard deviation/mean). The total bilirubin (TBIL) was implemented on the CL as a linear function (CL = CL_typical_ × ((TBIL-10.65) × −0.0107)). SIR: Sampling importance resampling approach. The uncertainty was derived from the SIR with 2,000 samples and 1,000 resamples.

### *In Silico* Simulation

**FIGURE 3:**
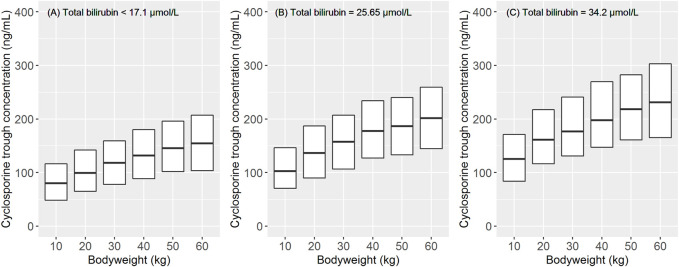
Impact of body weight and total bilirubin level on the pharmacokinetic exposure at steady state after the oral administration of cyclosporine A (5 mg/kg/d). The data of 1,000 children were used for the simulation for each body weight band. The simulation was stratified by different total bilirubin levels: **(A)** the normal level (<17.1 μmol/L). **(B)** and **(C)** 1.5 and 2 times the upper limits of the normal level (25.65 and 34.2 μmol/L), respectively. The total simulated exposure was presented as median values (25^th^–75^th^ percentiles).

After orally administering a 5 mg/kg daily dose of CsA, the model predicted a trough concentration at a steady state by body weight and TBIL levels, as shown in Figure 3. Considering the proposed dosing regimen, the exposure in pediatric patients with low body weight bands (<30 kg) was below the therapeutic windows (100–200 ng/ml), suggesting that an increase in dosage must be considered to achieve sufficient exposure. Moreover, pediatric patients with higher TBIL levels appeared to have higher exposure, and dose reduction in these patients was deemed necessary.

## Discussion

This is a pooled population PK analysis of CsA in pediatric patients with acquired AA in two study hospitals. The proposed population PK model could accurately describe the PK properties of CsA in the target population, and TBIL could affect clearance.

As CsA has high variability in PK profiles, routine TDM is mandatory in clinical practice for individual therapy. The population PK approach combined with Bayesian estimates for individual PK parameters offers a powerful tool to achieve this purpose. Regarding the structural model in population analyses for CsA, the one-compartment deposition model was commonly used for sparse PK data (Xiaoli and Qiang, 2009; Ni et al., 2013; Li et al., 2019; Albitar et al., 2020). The current modeling analysis using CsA trough and peak concentrations suggested an appropriate two-compartment disposition model, which was consistent with several published CsA population analyses (Wilhelm et al., 2012; Okada et al., 2017). In the current investigation, the apparent clearance (CL/F) estimate for a typical 28 kg child was 29.1 L/h, which was higher than that reported in a previous study including 102 children with AA (15.1 L in a child weighing 29.8 kg) (Ni et al., 2013). The discrepancy in CL/F might be attributed to the difference in sampling strategy (trough and peak concentrations in our study) and the sequential utilization of different structural models. However, the CL/F estimate in the present study was within the range of that of published population PK models for pediatric patients receiving transplantation. The CL/F was 23.1 L/h in 98 pediatric renal transplant patients weighing 35.2 kg (Irtan et al., 2007) and 29.3 L/h in 17 pediatric patients receiving stem cell transplantation with an average body weight of 32.4 kg (Willemze et al., 2008). In the present study, the central volume (Vc/F) estimate was 325 L for a typical 28 kg child, which was higher than that reported in a previous pediatric AA study (89.1 L for a 29.8 kg child) (Ni et al., 2013), pediatric patients who received renal transplants (70.3 L for a 35.2 kg child) (Irtan et al., 2007), and pediatric patients who received stem cell transplantation (42.7 L for a 32.4 kg child) (Willemze et al., 2008) and lower than that reported in a recent study of Chinese pediatric patients with nephrotic syndrome (2320 L for a typical 25 kg child) (Zhao et al., 2022). The Vc/F estimate was still within the range of reported values from literature. Moreover, the total V/F (Vc + Vp) normalized to a 70 kg adult was 1,468 L, which was similar to those reported in some studies with adult patients (1,080 and 1,010 L for Chinese adults (Zhou et al., 2012; Wang et al., 2022), and 1,990 L for Korean adults (Ji et al., 2011)).

Considering the principles of allometry in pediatrics, the body weight was highly suggested to be included in the model (Holford et al., 2013). Fanta et al. (2007) conducted a population PK analysis for 162 pediatric patients before a transplant and found that young patients (<8 years) had approximately 25% higher body weight normalized clearance than older children. In a population PK study of pediatric patients with acquired AA, the body weight was found to correlate with CL/F and V/F (Ni et al., 2013). In the present study, we applied the body weight value in all the clearance and volume parameters as an allometric function with the fixed exponent of 0.75 and 1.0, respectively, which did not result in a poor model. In addition, a few studies included body surface area (BSA) (Okada et al., 2017) in the model; however, this variable was not investigated in the present study since some height data were missing, and we were unable to calculate the BSA.

Since CsA undergoes liver metabolism and renal elimination, laboratory tests for liver and renal function show that PK disposition is seemingly affected in the body. Fanta et al. (2007) suggested that total plasma cholesterol level was correlated with CL/F in pediatric patients undergoing renal transplantation, with a 5.4% reduction per 1 mmol/L increase in cholesterol level. A population PK analysis of Chinese patients who underwent allogeneic hematopoietic stem cell transplantation showed that the plasma albumin level was inversely correlated with CL/F, with a 2.89% drop per 1 g/L increase in albumin level (Zhou et al., 2012). In the present study, total plasma cholesterol level was not routinely measured during outpatient visits; therefore, this covariate was not investigated. Moreover, we had a similar finding on the albumin covariate, but considering the poor precision of the estimate (>50%), the albumin value was removed from the final model. We identified TBIL as a significant covariate on CL/F, with a 1.07% reduction per 1 nmol/L rise in TBIL. Several studies on patients with transplantation have reported that TBIL, a biomarker of liver function, was relevant to CL/F (Wu et al., 2005; Ji et al., 2011). This finding suggested that dose adjustment may be required in patients with elevated TBIL levels.

Ni et al. (2013) found that SCr levels were a significant covariate on CL/F in pediatric patients with AA, with an 8.1% decrease per 1 μmol/L increase in SCr. Fanta et al. (2007) indicated that SCr levels were significantly correlated with CL/F in children undergoing renal transplantation, although the covariate effect size was small. A population PK analysis of CsA in Chinese pediatric patients receiving hematopoietic stem cell transplantation suggested a nonlinear relationship between estimated glomerular filtration rates and CL/F, with an exponent of 0.545 in power function (Li et al., 2019). In the present study, we did not identify a significant effect of SCr levels; the most likely explanation was that the majority of children had normal renal functions during the treatment period. Moreover, CsA was highly bound to erythrocytes and plasma proteins, and its distribution in the blood was reported to be approximately 41–58% in erythrocytes (Han et al., 2013). HCT was considered a significant covariate in a few previous studies (Wu et al., 2005; Yin et al., 2006; Fanta et al., 2007; Zhou et al., 2012). However, we did not find such a relationship in this study. Considering transfusion was needed in some patients, the relationship between Hb and Vc/F has been assessed with no statistical significance in the present study. However, a power relationship was suggested, with the exponent estimate of 0.159. Again, the Vc/F estimate was 290 L for a typical child with a Hb level of 85 g/L. The impact of Hb on Vc/F was not substantial.

Concomitant drugs, such as anabolic steroids (Ni et al., 2013) and triazole antifungal agents (Zhou et al., 2012; Li et al., 2019; Ling et al., 2021), have been reported to affect CsA PK exposure. Children undergoing IST often receive steroids as a concomitant drug (Ettenger, 1998; Benfield et al., 1999; Clucas et al., 2019). In theory, steroids could reduce CYP450 3A metabolism in CsA *via* competitive inhibition (Nakamura et al., 2002). The effects of steroids (methylprednisolone, prednisolone, or prednisone) on CsA PK exposure were further assessed in adult patients (Lam et al., 2008). In the present study, five types of steroids (Table 1) were comedicated with CsA in the treatment; however, the most frequently used steroids (testosterone, prednisone, and methylprednisolone) did not significantly influence CL/F according to the modeling analysis. Moreover, only a few patients (<5%) received triazole antifungal agent treatment, and its effect on CL/F was not further investigated.

This study has several limitations. 1) The CsA data were retrospectively collected from two centers, and a prospective clinical study would improve the data accuracy. 2) Enzyme polymorphisms have been demonstrated to contribute to the PK variability in CsA. However, relevant CYP3A and ABCB1 polymorphisms were not detected in this study, which may reduce the chance of finding polymorphism-related covariates. 3) In the present study, only two covariates were included in the final model; other significant covariates such as albumin were not retained due to the poor precision of parameter estimation. This model should, in the future, be updated with emerging data, which will allow the assessment of the covariates in a broader population and improve the goodness of fit accordingly, especially for population/individual predictions versus observation plots.

## Conclusion

In this study, we developed a population PK model to describe the PK property of CsA in Chinese pediatric patients with acquired AA. Body weight and TBIL level were significant covariates for the PK disposition of CsA. The proposed model could inform precision medicine in CsA therapy for pediatric patients with acquired AA.

## Data Availability

The original contributions presented in the study are included in the article/Supplementary Materials; further inquiries can be directed to the corresponding authors.
